# Anticipation in turn-taking: mechanisms and information sources

**DOI:** 10.3389/fpsyg.2015.00089

**Published:** 2015-02-02

**Authors:** Carina Riest, Annett B. Jorschick, Jan P. de Ruiter

**Affiliations:** Faculty for Linguistics and Literary Studies, Bielefeld UniversityBielefeld, Germany

**Keywords:** turn-taking, timing, anticipation, reaction, conversation

## Abstract

During conversations participants alternate smoothly between speaker and hearer roles with only brief pauses and overlaps. There are two competing types of accounts about how conversationalists accomplish this: (a) the signaling approach and (b) the anticipatory (‘projection’) approach. We wanted to investigate, first, the relative merits of these two accounts, and second, the relative contribution of semantic and syntactic information to the timing of next turn initiation. We performed three button-press experiments using turn fragments taken from natural conversations to address the following questions: (a) Is turn-taking predominantly based on anticipation or on reaction, and (b) what is the relative contribution of semantic and syntactic information to accurate turn-taking. In our first experiment we gradually manipulated the information available for anticipation of the turn end (providing information about the turn end in advance to completely removing linguistic information). The results of our first experiment show that the distribution of the participants’ estimation of turn-endings for natural turns is very similar to the distribution for pure anticipation. We conclude that listeners are indeed able to anticipate a turn-end and that this strategy is predominantly used in turn-taking. In Experiment 2 we collected purely reacted responses. We used the distributions from Experiments 1 and 2 together to estimate a new dependent variable called Reaction Anticipation Proportion. We used this variable in our third experiment where we manipulated the presence vs. absence of semantic and syntactic information by low-pass filtering open-class and closed class words in the turn. The results suggest that for turn-end anticipation, both semantic and syntactic information are needed, but that the semantic information is a more important anticipation cue than syntactic information.

## INTRODUCTION

Participants in a conversation have a number of tasks that they have to perform simultaneously. They have to comprehend the speaker’s utterance while at the same time they need to prepare their response to that utterance, preferably before the current speaker ends their turn. Despite the complexity of these processes the alternation between the speaker and the hearer roles is generally timed with only short pauses and overlaps (Sacks et al., 1974). This conversational phenomenon is an important part of the turn-taking organization.

There are two competing main approaches providing an explanation for the turn-taking organization: the *anticipatory* approach, in which it is assumed that participants are able to predict the end of a turn in advance, and the *signaling* approach, which assumes that listeners perceive specific signals to detect the end of a turn.

The aim of this study was first to determine the relative contribution of these two proposed mechanisms to turn-taking and second, to investigate which linguistic information sources listeners predominantly use for end-of-turn anticipation. To this end, we conducted a series of button-press experiments with turns from natural conversations while manipulating both the respective critical information sources and the task.

The anticipatory approach argues that the precise timing in conversations can only be explained by the listeners’ ability to make accurate predictions about the end of the speaker’s utterances. Depending on the assumed anticipatory model listeners use various kinds of information to anticipate. The first to claim that listeners are able to anticipate a turn ending were Sacks et al. (1974). In their famous and often-cited turn-taking model they provide an explanation for the characteristic smooth speaker transitions in natural conversation. According to their model, turns consist of syntactic building blocks called turn-constructional units. Listeners are able to predict the end of a turn-constructional unit. At this point a speaker change becomes relevant. This point in time is called a transition-relevance place. When a turn arrives at a transition-relevance place it is possible (a) for the current speaker to select another speaker, or (b) for another speaker to self-select and start talking. If neither option (a) nor (b) is used the current speaker can produce another turn.

In contrast, the signaling approach assumes that turn transitions are regulated by an exchange of conventional vocal or gestural signals (e.g., Yngve, 1970). So in this approach, participants in a conversation do not *anticipate* these signals but *react* to them after having perceived them. Influential proponents of the signaling approach who did numerous studies on finding explicit turn taking signals are Duncan (1972, 1973), Duncan and Niederehe (1974), and Duncan and Fiske (1977). They assume that there exist definite signals that are displayed and responded to according to specific rules. According to Duncan (1972) such signals are composed of one or more of six behavioral cues: (1) any phrase-final intonation other than sustained, intermediate pitch level, (2) drawl on the final syllable or on the stressed syllable of a terminal clause, (3) the termination of any hand gesticulation, (4) sociocentric sequences (stereotyped expressions like “you know,” “isn’t it,” etc.), (5) drop in pitch and/or loudness in conjunction with one of the sociocentric expressions, or (6) termination of a grammatical clause. According to Duncan and Fiske (1977) speakers always produce at least one of these turn transition cues at the end of their turn, to which listeners react by initiating their next turn. The more cues a speaker produces the more likely a change of speaker role is at that point.

The standard argument against the signaling approach is that the relevant cues occur too late in the speaker’s turn to enable timely speaker changes. As a counter-argument, Heldner and Edlund (2010) note that the timing of floor changes is not as precise as it is often claimed. In their analysis of three different conversational corpora 41–45% of between-speaker intervals were longer than 200 ms. They claim that these intervals are potentially long enough for people to react to end-of-turn signals. Their argumentation is based on the distribution of observed delays and pauses in conversational turn-transfers. In their view, pauses longer than 200 ms could also plausibly be explained by assuming they were reactions to signals (p. 566), while pauses shorter than 200 ms could correspond to anticipation (55–59% of the turn transitions in the investigated corpora). Their reaction threshold explanation is based on minimal response times, which were investigated under maximally favorable conditions. Their argument for this strict threshold is that interlocutors are highly trained to recognize gaps, when they can start their turn. But even if one assumes higher thresholds reaching up to 600 ms (Jescheniak et al., 2003; Indefrey and Levelt, 2004; Schnur et al., 2006) Heldner and Edlund (2010) argue that the proportion of responses which can be explained by reaction would be lower, but would not be eliminated.

We want to suggest that the presence of gaps longer than 200 ms does not necessarily mean that the turn before the gap was reacted to. Speakers often intentionally delay the production of so-called ‘dispreferred’ responses, which leads to longer pauses (see, e.g., Levinson, 1983; Kendrick and Torreira, 2014). So pauses longer than 200 ms are not necessarily caused by reaction, but can also be caused by an anticipated response that was nevertheless intentionally delayed. Conversely, response times of shorter than 200 ms need not always be caused by anticipation, but can be early reactions to perceived signals (false alarms). Hence, using a fixed cut-off value does not give us an accurate estimate of the relative number of anticipated and reacted turn transitions.

One possible criticism regarding the anticipatory approach is that Sacks et al. (1974) do not explain the mechanism responsible for anticipation, and more specifically, which information listeners use to ‘project’ when a turn is going to end (Sacks et al., 1974; Power and Dal Martello, 1986; O’Connell et al., 1990). Sacks et al. (1974) present only observational evidence suggesting that syntax and intonation play an important role in this process. But in the last decade possible mechanisms of turn-end anticipation have been investigated in more depth.

To investigate the role of intonational contour and lexico-syntactic cues in end-of-turn anticipation De Ruiter et al. (2006) performed a button press experiment presenting turns taken from natural Dutch conversations to participants. The instruction was to press a button when they thought the turn was going to end. They presented unaltered turns as well as manipulated turns where the lexico-syntactic information was absent but the intonational contour remained intact and vice versa. The intonational contour was manipulated by completely flattening the pitch leaving duration, rhythm and intensity intact. The lexico-syntactic information was manipulated by low-pass filtering the original turn fragment. In this way, words could no longer be identified, but the pitch contour remained intact. The results show that for unaltered turns, the average response time was about 200 ms before the turn was finished. This indicates that rather than waiting for the end of the turn and then react, the participants tried to anticipate the turn ending. With intonation contour absent but intact lexico-syntactic information, the participants were still able to accurately anticipate the turn ending. But the anticipation accuracy deteriorated significantly in absence of the lexico-syntactic information. The authors concluded that the lexico-syntactic structure is necessary (and perhaps even sufficient) for accurate end-of-turn projection. They suggested that the syntactic structure provides constraining information about the upcoming words and serves as a temporal resource for the listeners to monitor the unfolding turn. An important difference between the task used by De Ruiter et al. (2006) and turn-taking in natural communication is that listeners do not need to prepare and produce an utterance. This actually led to more accurate responses in the experiment compared to the responses in the natural conversations from which the experimental stimuli were culled. Hence, we believe that the results from this methodology are at least qualitatively generalizable to the natural situation.

Keitel et al. (2013) used eye-tracking methodology to investigate the influence of semantic content and intonation on anticipation ability during development. They presented recordings of actors performing conversations to three different age groups (prelinguistic 6–12 months, linguistic 24–36 months, adults) while measuring their gaze. The conversations were presented either with normal or flattened intonation. If a gaze was shifted from the current to the next speaker at least 500 ms before the end of the current turn, it was considered anticipatory. But if the gaze shifted after the listener began to speak the gaze shift was coded as reactive. The results showed that in contrast to younger infants, children at the age of three are already able to reliably anticipate the end of turns. Furthermore, intonation influenced anticipation only in this specific age group, suggesting that at that age they rely more strongly on intonational information for anticipation than adults. The authors explained this finding by noting that the syntactic and semantic competence of the 3-year-olds is not yet adult-like. This is in line with the finding that adults tend to rely on prosody for the detection of turn-ends only when neither semantic nor syntactic information is available (Grosjean and Hirt, 1996).

A comparable study was done by Casillas and Frank (2013) who also investigated which linguistic cues children use to anticipate a turn ending. In contrast to Keitel et al. (2013) they tested 1–7 year-olds and instead of using conversations done by actors, they measured the children’s gaze shifts while watching videos of conversations between puppets. Casillas and Frank (2013) found that even 1 and 2-year-olds anticipated turn endings, and that their anticipation correlated positively with the duration of the gap between two successive turns. They also manipulated the prosodic or lexical information (or both) of the conversations, and compared question with non-question turns. In their general discussion, they write that “Question effects are strongest when *both* prosodic and lexical cues are present, contrary to prior findings with adult listeners that found lexical information sufficient to predict upcoming turn-end boundaries (De Ruiter et al., 2006)” (emphasis in original). We are not convinced that there is a clear contradiction between their study and the result of De Ruiter et al. (2006) for the following reasons. First, the study by Casillas and Frank (2013) does not provide enough information to assess whether there is a statistically significant effect corresponding to this specific claim. Second, in the study by De Ruiter et al. (2006), the factor Question vs. No-Question was not investigated. (In Stivers et al. (2009) the data from De Ruiter et al. (2006) was reanalyzed and indeed showed no difference between responses to questions and non-questions, but that was only for the natural data.) Finally, it is possible, perhaps even plausible, that asking actors to record a conversation speaking “as if they were on a children’s television show” (p. 2) will result in prosodic patterns that are more exaggerated than in natural speech, due to the explicit child-directedness of the actors’ speech. For these reasons, we do not (yet) see a clear contradiction between the results of Casillas and Frank (2013) and those of De Ruiter et al. (2006).

To investigate how listeners use lexico-syntactic information to anticipate turn-ends Magyari and De Ruiter (2012) conducted a gating study. They used the experimental stimuli of De Ruiter et al.’s (2006) study and selected turns of which the ends were either predicted with a high or with a low accuracy in the button-press experiment. The results showed that the proportion of the correct guesses of upcoming words was higher when the accuracy of button-press in the original experiment was higher. Furthermore, in the gating study the participants expected more words to come with those turns that resulted in button presses that occurred too late in De Ruiter et al.’s (2006) study. They concluded that listeners make predictions in advance about *which*, and therefore *how many*, words will follow in a turn. These predictions help to estimate the remaining duration of the turn.

The idea that lexico-syntactic information serves as source for listeners’ anticipation performance is also supported by conversation-analytic studies (e.g., Ford and Thompson, 1996; Selting, 1996; Caspers, 2003). Caspers (2003) showed in her quantitative investigation that turn transitions are always located at syntactic completion points. She concluded that syntax constitutes the main information source for end-of-turn projection. Similar findings, based on a quantitative analysis of standard German, have been presented in Selting (1996), who concluded that listeners primarily exploit syntactic structure to project turn endings. Ford and Thompson (1996) found in their analysis of an American English face-to-face corpus that speaker change most frequently occurred when syntactic completion was combined with intonational as well as pragmatic completion. They concluded that syntax operates together with intonation and pragmatics to project the end of turns (see also Gravano and Hirschberg, 2011). As not all these studies found a perfect correspondence of syntactic completion points to turn-transitions, it remains an intriguing question how the distinction between those syntactic completions that are, and those that aren’t treated as turn-ends by the listeners is made. Unfortunately, this question cannot be satisfactorily answered by studying correlations in dialog corpora, but would require explicit experimentation to be able to distinguish correlation from causation.

To summarize, there is evidence from multiple sources that listeners are able to anticipate the end of the speaker’s turn (De Ruiter et al., 2006; Casillas and Frank, 2013; Keitel et al., 2013). But the mere existence of an anticipation ability does not imply that it is actually used to predict when a turn is finished in natural communication. Furthermore, Heldner and Edlund (2010) argued that turn-taking could at least partially be explained by assuming that conversationalists simply react to signals. Thus, the first question we want to investigate in this study is: is turn-taking based on anticipation or on reaction?

## EXPERIMENT 1

To determine the relative role of anticipation and reaction in turn-taking we conducted a button-press experiment using the same experimental methodology as in De Ruiter et al. (2006). We took turns from natural conversations and asked the participants to indicate the end of the turn by pressing a button. In the turns, we manipulated the information available for anticipation of the turn end and studied the effect of this manipulation on the projection accuracy. Our manipulations ranged from providing complete advance information about the turn-end to completely removing all linguistic information from the turn. (These manipulations are described in detail below.) The logic is that if the projection accuracy in responding to the original (unchanged) turns is comparable to responses to turns with advance information, then this is evidence for anticipation. On the other hand, if the projection performance to the natural turns is similar to the responses to the turns without or with substantially reduced linguistic information, this is evidence for people reacting to the perceived end of the turn.

## MATERIALS AND METHODS

### Compliance with ethics guidelines

The experimental methods used in this project have been approved by the Ethics Board of Bielefeld University. Informed consent was obtained from all subjects.

### Participants

Eighty native speakers of German participated in Experiment 1 (56 females, 24 males).

### Stimulus collection

The stimulus collection procedure is the same as the one described in De Ruiter et al. (2006). For maximum ecological validity we took our stimuli from a natural German ‘telephone’ corpus (audio-only conversation), which we recorded in our lab. We recorded 16 native speakers of German in eight dyadic conversations (four female–male, three female–female, one male–male). The participants in each dyad were friends. For the stimulus collection we told the participants to just talk about anything they liked and gave them no further instruction. Each dyad’s conversation lasted 20 min, resulting in a total of 160 min of recorded conversation.

For the audio recordings we put the participants in two separate rooms and required them to wear closed headphones. Directional microphones were placed on a table in front of them. We established a telephone-like connection between them, such that both participants could hear both themselves and their interlocutor. The speech of each of the two participants was recorded separately on the two channels of a stereo recording device. This way, we avoided cross talk between the participants in our recordings. The participants rapidly got used to the recording situation and the resulting conversations appeared natural and lively.

**FIGURE 1:**
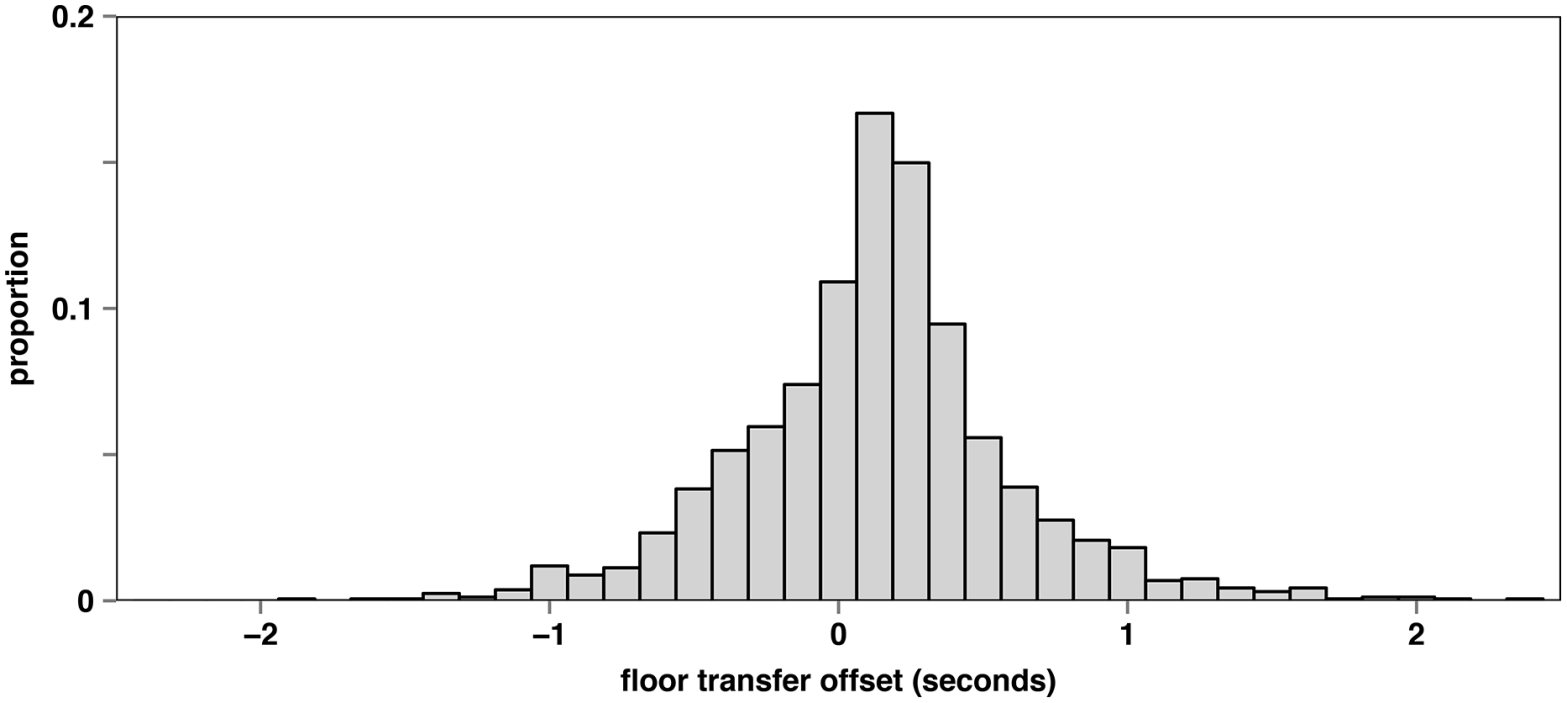
**Floor Transfer Offset (FTO) distribution of the German telephone corpus**.

After recording the corpus, the conversations were transcribed, registering overlaps, pauses, laughter, turn beginnings and endings, assessments (Goodwin, 1986), and continuers (Schegloff, 1982). In addition we measured the Floor Transfer Offset (FTO) of 1597 turn transitions. The FTO value is defined “as the difference (in seconds) between the time that turn starts and the moment the previous turn ends” (De Ruiter et al., 2006, p. 516). Hence, a gap between two turns is characterized by a positive FTO value and an overlap by a negative one. **Figure 1** shows the distribution of the FTO values.

Although the general shape of the FTO distribution resulting from the German telephone corpus looks similar to the Dutch FTO distribution from De Ruiter et al. (2006), the distributions differ in a number of aspects. There are small differences in the means, variances, skewness, and kurtosis (see **Table 1**) ^1^.

**Table 1:** Comparison of Dutch and German telephone corpora.

		Dutch telephone FTO	German telephone FTO
*N*		1507	1597
Mean	[ms]	0	131
Median	[ms]	38	141
Mode	[ms]	173	162
Variance	[ms]	338	234
Minimum	[ms]	-3080	-2955
Maximum	[ms]	2839	2902
Skewness		-0.348	0.136
Kurtosis		6.923	3.124

From this corpus we randomly selected 100 target turns and an additional 16 turns for practice purposes. We took care that the turns contained at least five words so that the participants in the planned button-press experiments obtained enough information content to potentially base their reaction on. Furthermore, we made sure that the random selection reflected the distribution of pauses and overlaps of the natural conversations. Furthermore we balanced the sex of the speaker in the target turns (50 % female, 50% male). The total number of different speakers in our target stimuli was 16. **Table 2** presents some descriptive statistics of the target turns.

**Table 2:** Descriptive statistics of target turns.

		Minimum	Maximum	Mean	Mode	SD
Duration	[ms]	863	7105	3157	3136	1415
FTO	[ms]	-1828	1257	96	-70	417
Number of Words		5	29	13	8	6

After selecting the target turns, we extracted them into individual sound files using Praat (Boersma and Weenink, 2012) and created four different versions of each stimulus. These versions were as follows.

***Natural-Turn**.* The target turn was presented as it occurred in the natural conversations. In this condition the participants had access to all potentially relevant information to base their anticipation orreaction on.

***Advance-Knowledge**.* The participants could first read the content (a literal transcription) of the turn before they heard the target stimulus. Because the participants knew in advance how the turn was going to end, they were, in principle, maximally capable to anticipate the turn end. In this condition the response distribution of anticipated responses was measured.

***Scrambled-Word-Order**.* We randomly changed the order of the words within the target turn using Praat. The pauses between the words in the original were assigned to the subsequent word. The resulting stimuli therefore had the same duration as the Natural-Turn stimuli. In this condition there was no sequential word-order information to base the anticipation on, but there were still words present. Thus, the predictability of a word on the basis of its preceding words is switched off, i.e., the cloze probability (Taylor, 1953) of the words in the resulting turns was very low. In contrast to the Natural-Turn condition the anticipation of the turn end on the basis of sequential lexical information was made impossible.

***Noise**.* The Noise condition was created using a Praat script that convolved the speech stimulus of the natural turn with white noise. The resulting sample of constant noise had the same duration and frequency spectrum as the original fragment. This condition served as a comparative baseline from which all linguistic information that could be used for anticipation was removed. The only way to be certain that the turn has ended in this condition is to react to the turn end. This condition measured the response distributions when the participants had no choice but to react to the end of the turn.

In order to control for subjective loudness between conditions and stimuli we adjusted the loudness of all stimuli to a reference sone value.

### Design

Each participant was presented with four trial blocks (Natural turn, Advance-Knowledge, Scrambled-Word-Order, Noise) each containing 25 target turns. Within each block there were four practice trials followed by the 25 target turns. We created eight different experimental lists. In the first four lists we permutated the block order according to a Latin-square design. The remaining four lists were the same as the first four lists with the block order as well as the presentation order of the stimuli reversed. Each of the target turns appeared in all four conditions across the lists but none appeared twice within the same experimental list.

### Procedure

The participants received a written instruction that they had to listen to short audio fragments, taken from real conversations, and to press a button as soon as they thought the speaker in the fragments would finish speaking. They were informed that they would be presented with four different blocks, and that in one of these blocks they had to first read the content of the fragment before they heard the corresponding audio fragment. Furthermore, they were informed that in two blocks the stimuli were manipulated acoustically. The stimuli were presented to them via closed headphones. We randomly assigned the participants to one of the eight experimental lists (10 per list).

The participants were presented first with the four practice trials and after that with the corresponding trial block. After each practice block the participants got the chance to ask the experimenter questions. Each experimental block contained a visual countdown from 3 to 1 followed by the auditory presentation of the stimuli. As soon as the participants pressed the button the sound was immediately cut off. In this way we made sure that the participants got no feedback about their performance. The trial block Advance-Knowledge differed from the other trial blocks because after the visual countdown the participants were presented with a written sentence, representing the content of the turn. After pressing the button the sentence disappeared and the acoustic presentation of the stimulus started.

For the presentation of the stimuli we used the E-Prime software package (Schneider et al., 2012a,b), which also allowed us to record the time from stimulus onset to button press.

### Results and discussion

**FIGURE 2:**
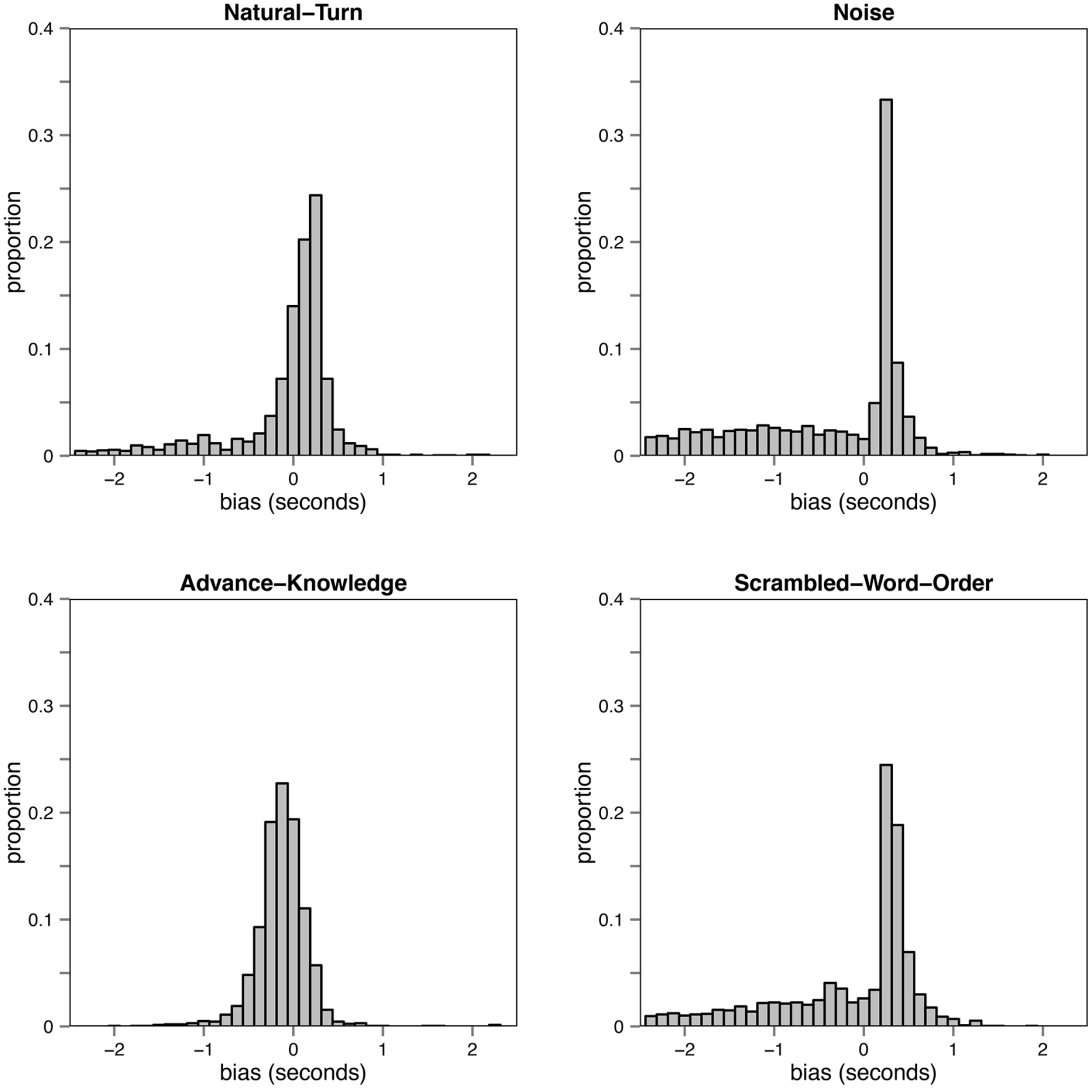
**Response distributions per condition from Experiment 1**.

**FIGURE 3:**
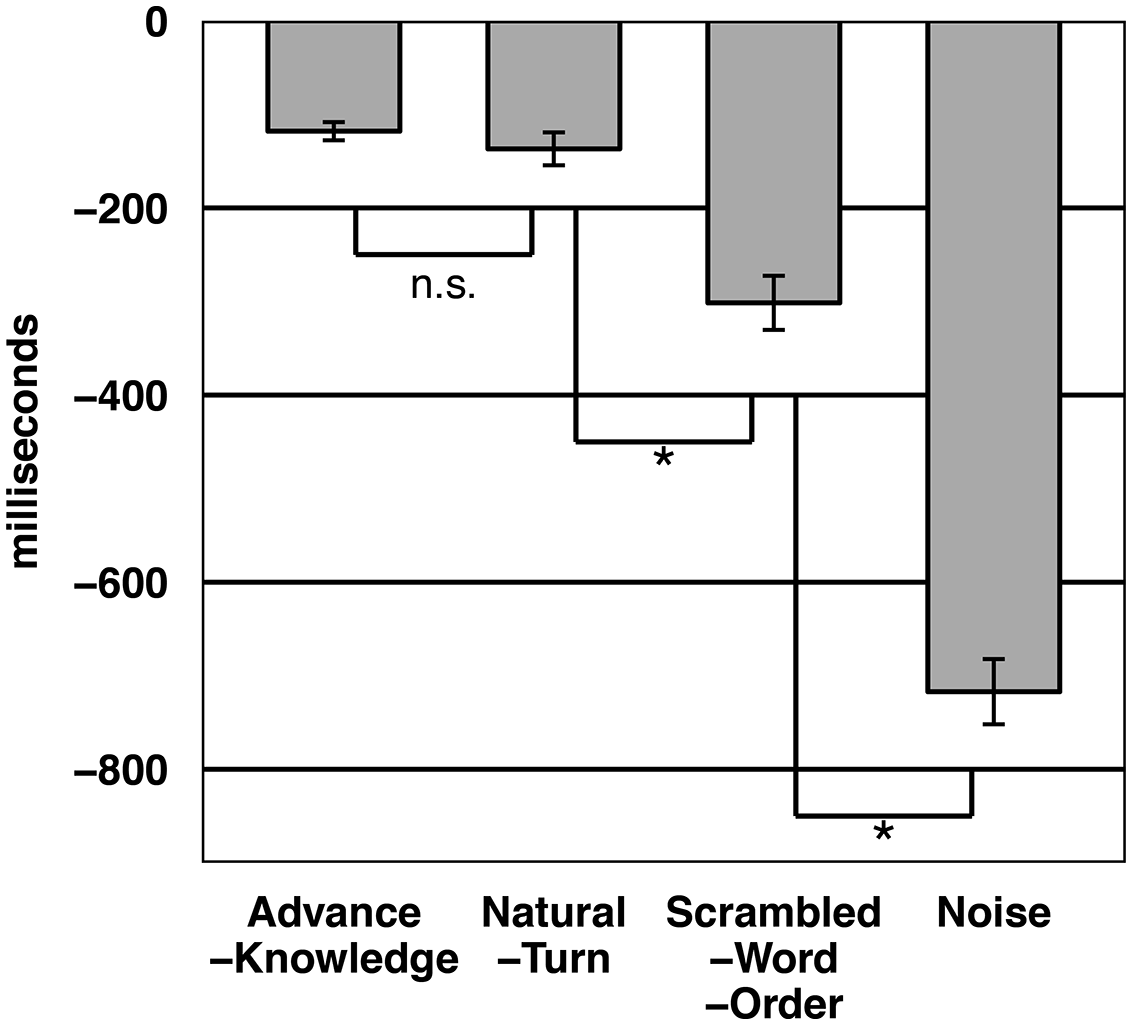
**Average BIAS of responses per condition as measured in Experiment 1.** Asterisk indicates statistical significance at the 0.05 level.

We first calculated the BIAS, which is defined as response time minus the duration of the target turn. **Figure 2** shows the BIAS distributions for the four different conditions. **Figure 3** shows an overview of the average BIAS per condition. The average BIAS is negative in all conditions, which gives a first hint that participants tried to anticipate the turn ending, rather than wait until the turn fragment was over.

An ANOVA for the dependent variable BIAS showed a significant effect for presentation condition (by subjects: *F1*(3,315) = 23.259, *p* < 0.001; by items: *F2*(3,297) = 18.82, *p* < 0.001). Bonferroni-corrected paired *t*-tests, pairing over identical turn fragments from the two conditions under comparison, revealed that the Natural turn condition led to significantly more negative BIAS than the Noise and the Scrambled-Word-Order condition. The latter condition led to significantly more negative BIAS than the Noise condition. Whereas the BIAS in the Advance-Knowledge and the Natural turn condition did not differ significantly from each other.

Conventional significance tests are designed to reject the null hypothesis without fault in the limit of infinite sample size. This is characterized by vanishing *p*-values and unbounded *t*-values. In contrast, if the null hypothesis is true and infinite sample sizes are considered the *p*-values are not converging to any limit value. Correspondingly, under the null hypothesis, all *p*-values are all equally likely (Rouder et al., 2009). Hence, it is not possible to claim evidence favoring a null hypothesis using conventional significance tests. We therefore also performed a Bayesian analysis (Jeffreys, 1961; Kass and Raftery, 1995) for the Advance-Knowledge and the Natural-Turn condition by comparing them using a Bayesian paired *t*-test (Rouder et al., 2009). To be consistent with Morey and Rouder (2011) and Rouder et al. (2012) we used a Cauchy prior with scale parameter for the standardized effect size in combination with a Jeffreys prior on the variance. The analysis was performed using the BayesFactor package (Morey et al., 2014) for R (R Development Core Team, 2009). An overview of a common textual interpretation of Bayes Factor values is presented in **Table 3**.

**Table 3:** Evidence Categories for Bayes Factor, adapted from Jeffreys (1961), cited in Wetzels et al. (2011).

Bayes factor	Interpretation
>100	Decisive evidence for H_A_
30–100	Very strong evidence for H_A_
10–30	Strong evidence for H_A_
3–10	Substantial evidence for H_A_
1–3	Anecdotal evidence for H_A_
1	No evidence
1/3–1	Anecdotal evidence for H_0_
1/10–1/3	Substantial evidence for H_0_
1/30–1/10	Strong evidence for H_0_
1/100–1/30	Very strong evidence for H_0_
<1/100	Decisive evidence for H_0_

The Bayesian paired *t*-test using item means for the variable BIAS revealed that the null hypothesis, stating that Advance-Knowledge and Natural-Turn condition are equal in anticipation accuracy, is twelve times more likely than the alternative hypothesis that these two conditions differ in button press accuracy (BF = 0.08). This provides “strong” evidence for the null hypothesis.

Comparing the subject means of the BIAS variable with the Bayesian paired *t*-test resulted in “substantial” evidence (BF = 0.1) for the null hypothesis. This analysis allows us to conclude that there is no statistically reliable difference between the BIAS in the Advanced-Knowledge and the Natural Turn condition. So the participants’ button press accuracy with the natural turns was just as good as when they had advance information about the content of the turn. This finding suggests that participants are indeed able to anticipate a turn ending, and that they are using this strategy to predict when a turn is going to end.

The significant difference between Scrambled-Word-Order and Noise condition indicates that having access to words (even though they were in the wrong order) still allowed them to anticipate better than chance.

Although there was no significant difference in the button press accuracy between the Advance-Knowledge and the Natural-Turn condition, the participants could still have reacted to signals to a certain extent. If the participants used both anticipation and reaction as a strategy this should result in a lower *response consistency*. To investigate the response consistency over conditions we computed the Entropy for every stimulus/condition pair (Shannon, 1948). The Shannon Entropy is a measure of uncertainty: the more the responses are distributed over different intervals the higher the Entropy. If the participants used only one strategy to estimate when the turn is over, the Entropy should be lower. However, if the participants used both reaction and anticipation, their responses should be more highly distributed, resulting in a higher Entropy.

**FIGURE 4:**
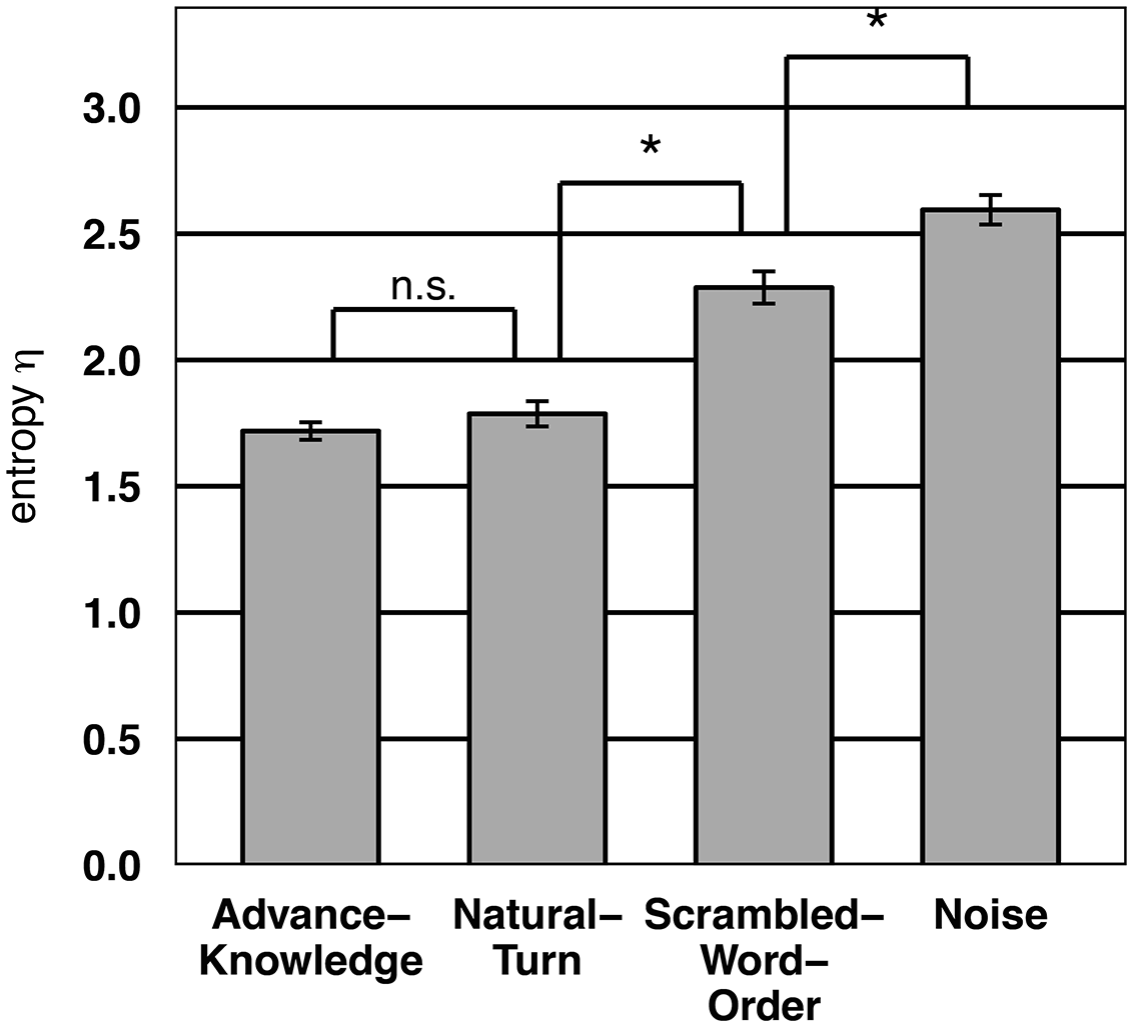
**Average Shannon Entropy per stimulus/condition as measured in Experiment 1.** Asterisk indicates statistical significance at the 0.05 level.

In **Figure 4** the average Shannon Entropy (using a bin-width of 250 ms; see De Ruiter et al., 2006 for details) is shown for every condition. We can only show a by-item analysis as these Entropy values can only be meaningfully computed for individual stimuli over entire response distributions.

As in the BIAS analysis, an ANOVA of the Entropy showed a main effect for condition *F2*(3,297) = 62.5, *p* < 0.001). Bonferroni-corrected paired *t*-tests revealed that all differences between individual conditions were significant (*p* < 0.001), the exception being the difference between Advance-Knowledge and Natural-Turn.

Again we compared the Entropy values in the Advance-Knowledge and Natural-Turn condition using a Bayesian paired *t*-test. The analysis (BF = 0.2) provided “substantial” evidence for the null hypothesis (no difference between Advance-Knowledge and Natural-Turn in button press consistency).

The analysis of the participants’ button press consistency supports the interpretation of the BIAS results. The results showed that the Entropy in the Natural-Turn condition and the Advance-Knowledge condition was comparable. Thus, in the Natural-Turn condition the participants were able to consistently and accurately anticipate the turn-end and consequently used anticipation as a strategy to tell when a turn was over.

In contrast, in the Scrambled-Word and the Noise condition the Entropy values were significantly higher than in the other two conditions. This suggests that the participants tried to anticipate the turn-end rather than just waited for the end of the fragment, which lead to significantly broader distributed responses. In addition, the average Entropy in the Scrambled-Word order condition was significantly lower than in the Noise condition. This corresponds to the BIAS analysis above where participants in the Scrambled-Words condition were significantly more accurate in detecting the turn end. Hence, the participants are more consistent and accurate in the end-of-turn projection when they have access to words compared to when they only hear noise. One explanation of this finding could be that even with the scrambled word order listeners are able to recognize the basic meaning of the turn, enabling them to roughly guess when the turn finishes. Additionally, it is possible that once the participants “gambled” that a certain word was the last word, they could anticipate the end of that word, as suggested by research on auditory word recognitions (Marslen-Wilson and Welsh, 1978; McClelland and Elman, 1986; Marslen-Wilson, 1987).

We showed in Experiment 1 that listeners in dialog are indeed able to anticipate the end of the speaker’s turn and that they consistently use this ability to predict when a turn is going to end. When listening to the natural turns the participants showed the same response accuracy and consistency as when they knew the end of the turn in advance. Our results are in line with earlier findings that listeners anticipate turn endings and that natural language is predictable to a certain degree (De Ruiter et al., 2006; Magyari and De Ruiter, 2012; Casillas and Frank, 2013; Keitel et al., 2013; Magyari et al., 2014). Hence, in the first experiment we were able to show that anticipation is the primary mechanism underlying smooth turn-taking, and that participants consistently use this strategy to detect a turn ending. Thus, our results support the turn-taking model proposed by Sacks et al. (1974). Nevertheless, reaction to the turn end might well serve as some kind of a “backup” mechanism in the case when the anticipation of the turn ending is, for whatever reason, not possible.

We now have an empirically derived distribution of anticipation times, from a task in which the participants were asked to anticipate turn-ends, and had the information to do so. To find out about the distributional properties of the reaction process, which we assume also plays a role, we need to study the reaction time distribution of participants that had no information to anticipate (as in the Noise condition of Experiment 1) but in addition, were not instructed to anticipate, but rather to respond to the end of the stimulus. To this end, we conducted Experiment 2.

## EXPERIMENT 2

Heldner and Edlund (2010) suggested that turn transitions with a gap longer than 200 ms are potentially explainable by assuming that participants respond to signals at the end of the turn. As we discussed in the introduction, this assumption does not capture the stochastic nature of the time course of the two processes involved. Instead, we assume that distributions of natural floor transfer are actually a stochastic mixture of an anticipation and a reaction time distribution. We wanted to empirically estimate the distribution of reacted responses in order to be able to estimate the proportion of turn-transitions that we were reasonably sure were reactions (and not to anticipations) to turn transitions.

An empirically estimated anticipation distribution is provided by the Advanced-Knowledge condition of Experiment 1. In Experiment 2 we want to find the other distribution based on pure reaction time. To this end, we used the Noise stimuli from Experiment 1, but now explicitly instructed the participants to respond only after they perceived the end of the fragment.

## MATERIALS AND METHODS

### Participants

Twenty native speakers of German participated in the second experiment (14 females, 6 males). None of the participants in Experiment 2 had taken part in Experiment 1.

### Stimuli and design

Each participant was presented with all of the 100 noise target stimuli created in Experiment 1. In addition we took four stimuli from the practice block for practice purpose. There were two experimental lists, whereas in the second list the presentation order of the stimuli (including the practice trials) was reversed.

### Procedure

The participants received a written instruction that they had to listen to noise fragments and press a button as soon as the noise stopped.

Within the experiment the participants were presented first with the four practice trials followed by the 100 target stimuli. After the practice trials the participants got the opportunity to ask questions. After the presentation of the first 50 target stimuli there was a break. The participants had to start the presentation of the remaining 50 stimuli by pressing a button, so that they could determine the length of the break by themselves. The participants were randomly assigned to one of the two experimental lists (10 per list).

### Results and discussion

**FIGURE 5:**
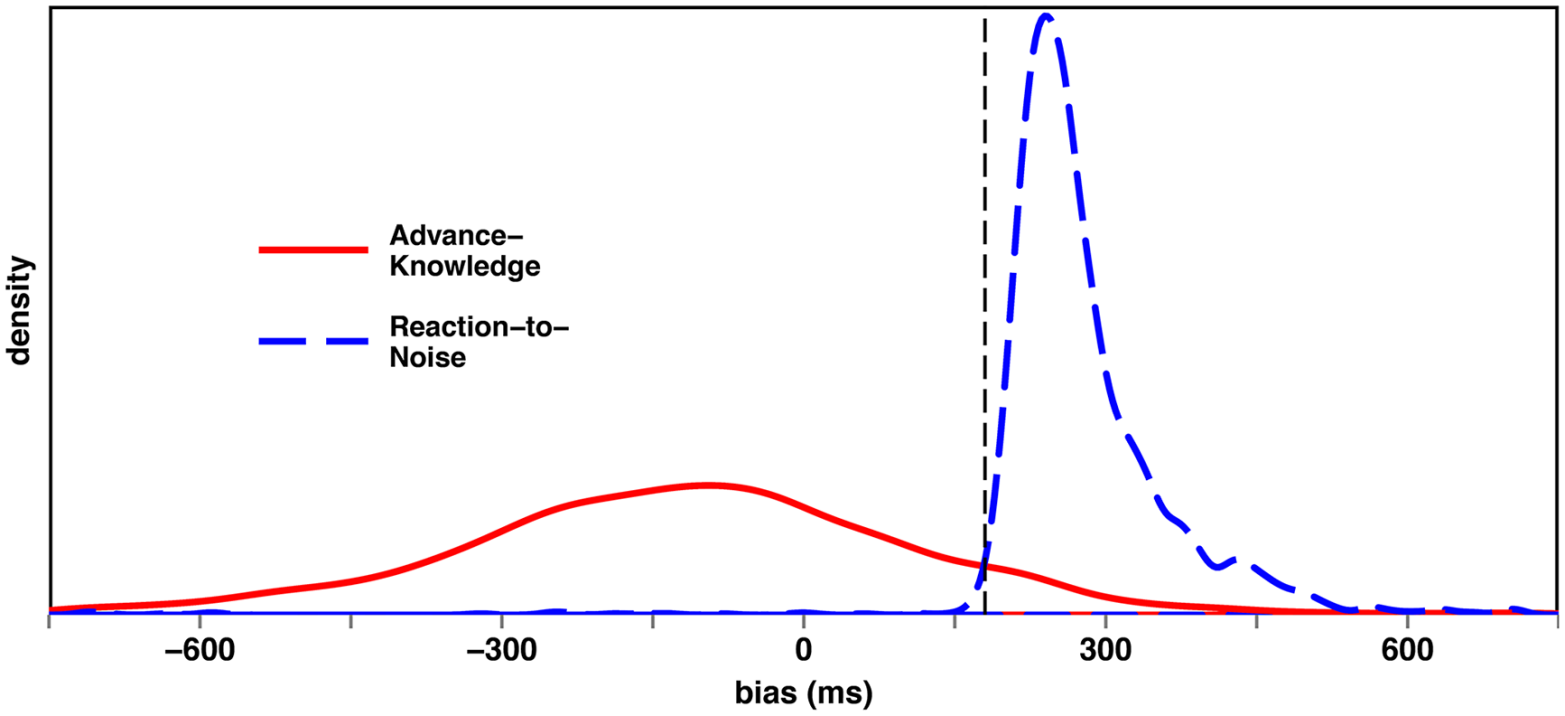
**Anticipation and reaction intervals**.

The reaction time distribution obtained in this experiment is presented together with the anticipation distribution from Experiment 1 in **Figure 5**. As expected, the reaction time distribution shows a pronounced sharp peak at a positive FTO (i.e., after the stimulus) whereas the anticipation distribution is broader and extends into the negative FTO range. In addition, the mode of the anticipation distribution is at a negative FTO value. The intersection of the two distributions characterizing the response time at which anticipation and reaction are equally probable is in good agreement with the 200 ms cut-off value proposed by Heldner and Edlund (2010). Nevertheless, the broad overlap of the two distributions shows clearly that the use of a categorical cut-off would not do justice to the stochastic nature of these two processes.

This is why we define a new measure designed to capture the *relative probability* of anticipation and reaction. The so-called Reaction Anticipation Proportion (RAP) value is defined as the natural logarithm of the ratio of anticipation and reaction probability.

(1) RAP(t)=log⁡e(PAnt(t)PRe⁡ac⁢(t))

Equation 1: Definition of the RAP value as logarithmized ratio of the anticipation P_Ant_(t) and reaction probabilities P_Reac_(t) at time *t*.

In Eq. (1) **P_Ant_(t)** and **P_Reac_(t)** denote the probability that a response at time *t* was an anticipation or reaction, respectively. These probabilities were computed in R (R Development Core Team, 2009) using the density distributions (cosine kernel and 2.5 Sheather and Jones (1991) bandwidth) from the Advanced-Knowledge condition of Experiment 1 and the Noise condition of Experiment 2. To account for noise in the data leading to possibly infinite RAP values we used a cutoff value of 10^-4^ in the factor calculations. Due to the log-scale of the RAP ratio negative values corresponds to a higher probability of reaction whereas a positive value indicates that anticipation is more likely.

**FIGURE 6:**
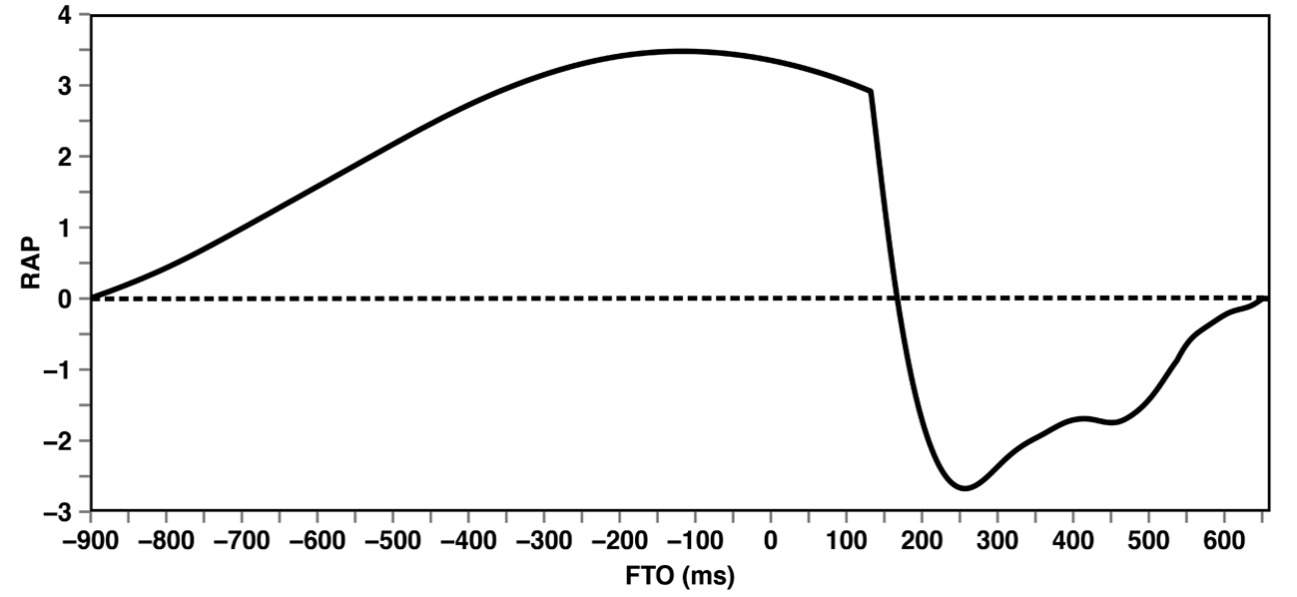
**Reaction Anticipation Proportion (RAP) value as a function of the FTO value**.

The RAP as a function of FTO is presented in **Figure 6**. The RAP is positive for a broad FTO interval ranging from about -750–200 ms and negative for FTO values in the interval from about 200–550 ms. Hence, reaction is more probable only in a relatively brief time interval. In addition, the influence of the pronounced sharp peak of the reaction distribution on the RAP value is weakened by the non-vanishing anticipation probability in the corresponding FTO range.

To demonstrate and validate the use of the RAP measure we applied it to the data analysis of the Natural turn and Noise conditions of the first experiment. The mean RAP value of the Natural turn condition was 0.60 and of the Noise condition -0.53. This supports our interpretation of the results of Experiment 1 that in the Natural turn condition the participants anticipated the end of the turn. In contrast, the responses in the Noise condition were predominantly based on reaction. It is noteworthy that the absolute value of the mean RAP of the two conditions are comparable, indicating that anticipation and reaction are about equally probable in the correspondingconditions.

We used the RAP measure to study the relative contribution of semantic and syntactic information in end of turn anticipation in Experiment 3.

## EXPERIMENT 3

In Experiment 3 we investigated the relative role of syntax and semantics as a cue for end-of-turn anticipation. Experimental as well as corpus-based studies (Grosjean and Hirt, 1996; Selting, 1996; Caspers, 2003; De Ruiter et al., 2006; Magyari and De Ruiter, 2012; Keitel et al., 2013; Magyari et al., 2014) suggest that lexico-syntactic information serves as the main information source for end-of-turn prediction. But in these studies semantic and syntactic information is confounded, so the relative role of the individual source of information in turn anticipation cannot be established.

To tease apart semantic and syntactic information in natural communication we used the widely used distinction between open and closed class words in linguistics and psycholinguistics. Open class words in German contain nouns, verbs, adjectives, adverbs, and proper names. The open class words are “rich in referential meaning” (Chiarello and Nuding, 1987, p. 539) and “are the main bearers of meaning in language, providing the building blocks for the overall sense that is contained in spoken and written sentence” (Brown et al., 1999, p. 261). New words are easily added to the item set and they constitute the main part of our vocabulary (Segalowitz and Lane, 2000). The closed class category in German contains prepositions, articles, conjunctions, pronouns, modal verbs, quantifiers, and particles. Closed class words are semantically empty and serve primary a syntactic role (Crystal, 1988, p. 37). They serve to build the “structural skeleton of the sentence” (Kedar et al., 2006, p.325) and bear solely grammatical information (Jakubowicz and Goldblum, 1995, p. 247). The closed class contains a specified set of items, in which the addition of new objects trough cultural change is very slow (Segalowitz and Lane, 2000). Although closed class words only form a minority of our vocabulary, they are used much more frequently than open class words (Baayen et al., 1995; Rochon et al., 2000). To summarize, “the distinction between open- and closed class words can be seen as a basic reflection of the separation between syntax and semantics” (Brown et al., 1999, p. 261) ^2^.

Therefore, in this experiment, we operationalized semantic information as open class words, and syntactic information as closed class words. To address the question which information source listeners use for anticipation, we conducted a similar reaction time experiment as in Experiment 1. We manipulated the presence of semantic and syntactic information in the turn fragments from Experiment 1 by acoustically manipulating the recognizability of open- and closed class words. To evaluate the influence of these manipulations on the anticipation and reaction probability we used the RAP measure introduced before.

If only syntax is used for end-of-turn prediction, then the absence of closed class words should result in a decrease in anticipated and an increase of reacted responses. On the other hand, if semantic information is used for the anticipation of a turn ending, we expect a deteriorated anticipation performance in absence of open class words. However, if both semantic and syntactic information are used to the same extent, then the effect should be similar in absence of content as well as closed class words.

### MATERIALS AND METHODS

#### Participants

Eighty native speakers of German who neither participated in Experiment 1 nor in Experiment 2 participated in Experiment 3 (53 females, 27 males).

#### Stimuli

The same 100 target and 16 practice turns as in Experiment 1 were used. We created four different versions of each turn fragment (see **Table 4** for an example of one experimental stimuli in all conditions). Natural Turn: the target turn was presented as it occurred in the natural conversation. Closed-Class-Words-Removed: the closed class words were “removed” by low-pass filtering (at 500 Hz Hanning Window). Open-Class-Words-Removed: by low-pass filtering we “removed” the open class words (at 500 Hz Hanning Window). Intonation-Only: the whole turn was low-pass filtered (at 500 Hz Hanning Window) so that no words could be recognized, but intonation remained intact. This condition served as a comparative baseline since neither syntactic nor semantic information were left in the turn fragment.

**Table 4:** Example of one experimental turn in all four conditions (underlined the respective low-pass filtered words).

Condition	Example
Natural-Turn	ich äh warte erstmal auf meine schwester und rufe die dann heute an
Closed-Class-Words-Removed	ich äh warte erstmal auf meine schwester und rufe die dann heute an
Open-Class-Words-Removed	ich äh warte erstmal auf meine schwester und rufe die dann heute an
Intonation-Only	ich äh warte erstmal auf meine schwester und rufe die dann heute an

In the conditions Open-Class-Words-Removed and Closed-Class-Words-Removed the number of filtered words were made equal to the minimum number of open class words and closed class words in the turn. In this way we made sure that the number of filtered open and closed class words were the same for the same source stimulus. The decision which words were low-pass filtered was randomized. In order to control for subjective loudness between conditions and stimuli we again adjusted the loudness of all stimuli to a reference sone value.

#### Design

Each participant in the experiment was presented with three trial blocks: (1) Natural-Turn, (2) Intonation-Only, (3) The stimuli from the Closed-Class-Words-Removed and Open-Class-Words-Removed condition. The latter were presented within one block. The blocks Natural-Turn and Intonation-Only contained 25 and the combined block Closed-Class-Words-Removed and Open-Class-Words-Removed 50 target turns (25 stimuli from Closed-Class-Words-Removed and 25 stimuli from Open-Class-Words-Removed). Within each block there were four practice trials followed by 25 and 50 target turns, respectively. We created eight experimental lists. As in Experiment 1, we permutated the block order in four of these lists according to a Latin-square design. The remaining four lists were the same as the first four lists with the presentation order of the target stimuli and the practice trials reversed. As in Experiment 1 the lists were constructed so that all of the 100 target stimuli appeared in all four conditions across the lists but none appeared twice within the same experimental list.

#### Procedure

We used the same procedure as in Experiment 1.

#### Results and discussion

**FIGURE 7:**
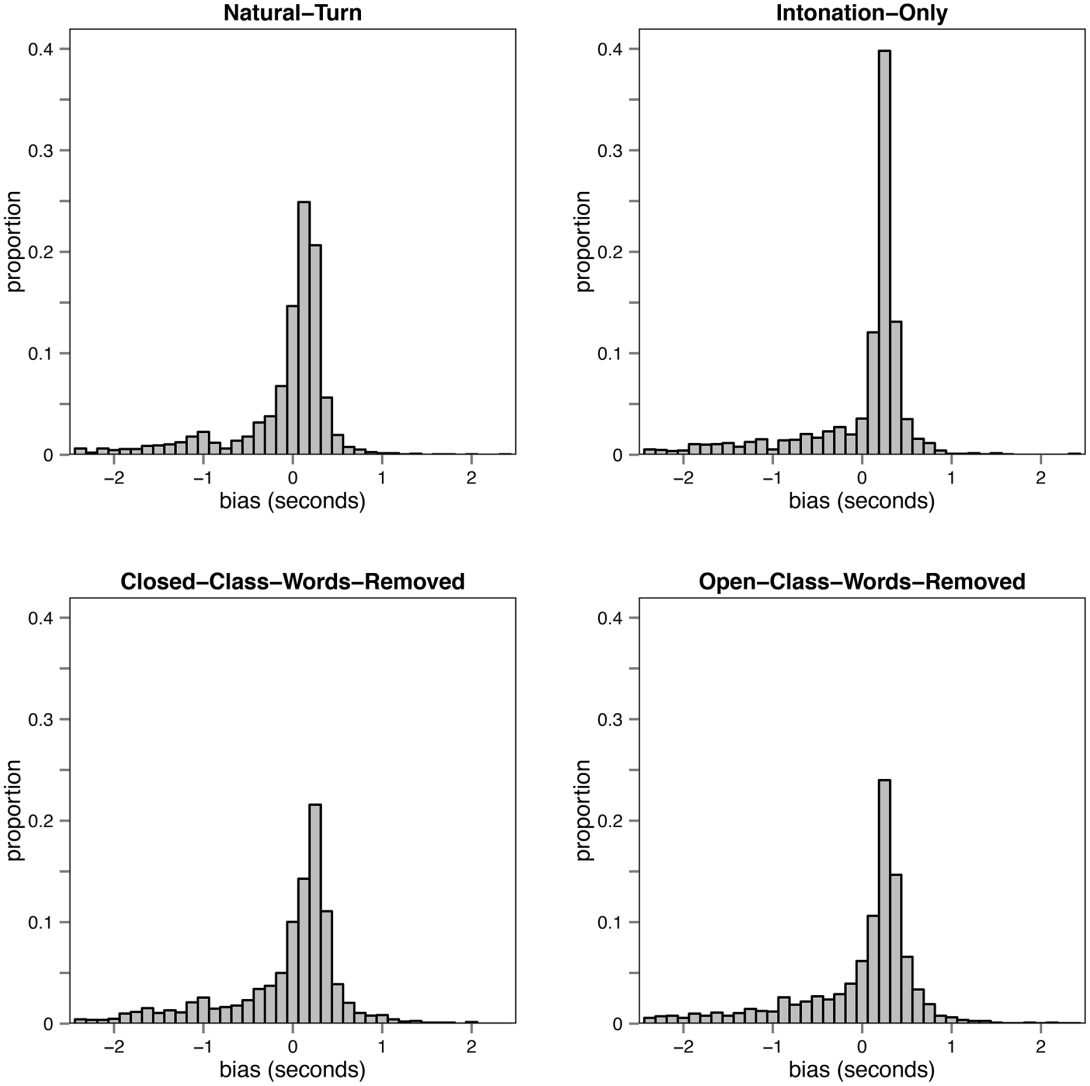
**Response distributions per condition from Experiment 3**.

**FIGURE 8:**
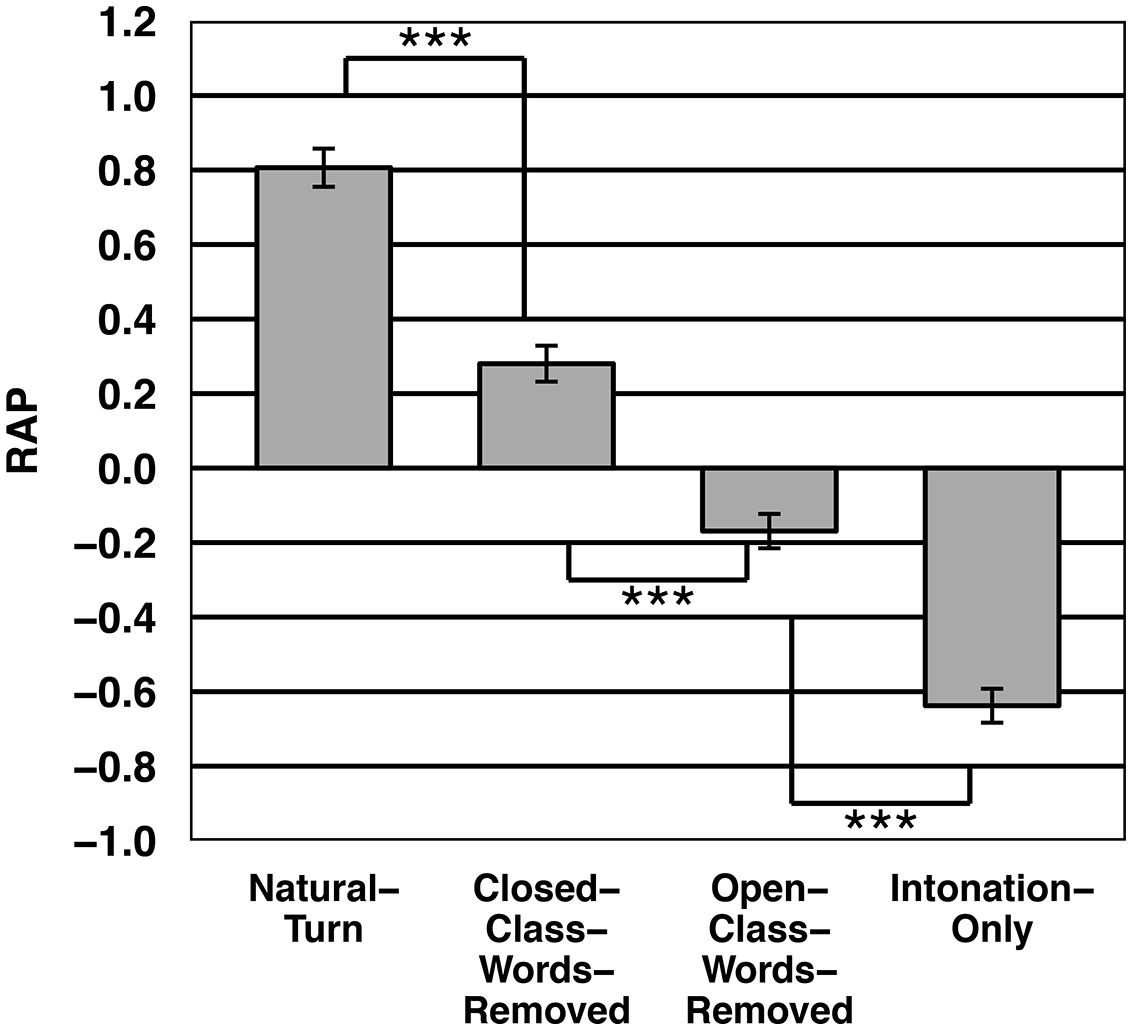
**Average RAP value per condition from Experiment 3.** Asterisks indicate statistical significance at the 0.001 level.

**Figure 7** shows the response distributions for the four different conditions. **Figure 8** shows the average RAP values for the different conditions. The average positive RAP values in the Natural-Turn and the Closed-Class-Words-Removed condition indicate that the participants anticipated more frequently than reacted to the end of the turn in these conditions. In the Open-Class-Words-Removed and the Intonation-Only condition the participants reacted more often to the end of the turn.

An ANOVA on the RAP values showed a significant effect for presentation condition (by subjects: *F1*(3,315) = 47.85, *p* < 0.001, by items: *F2*(3,297) = 74.11, *p* < 0.001). Bonferroni-corrected paired *t*-tests revealed that all differences between individual conditions were significant (*p* < 0.001).

**FIGURE 9:**
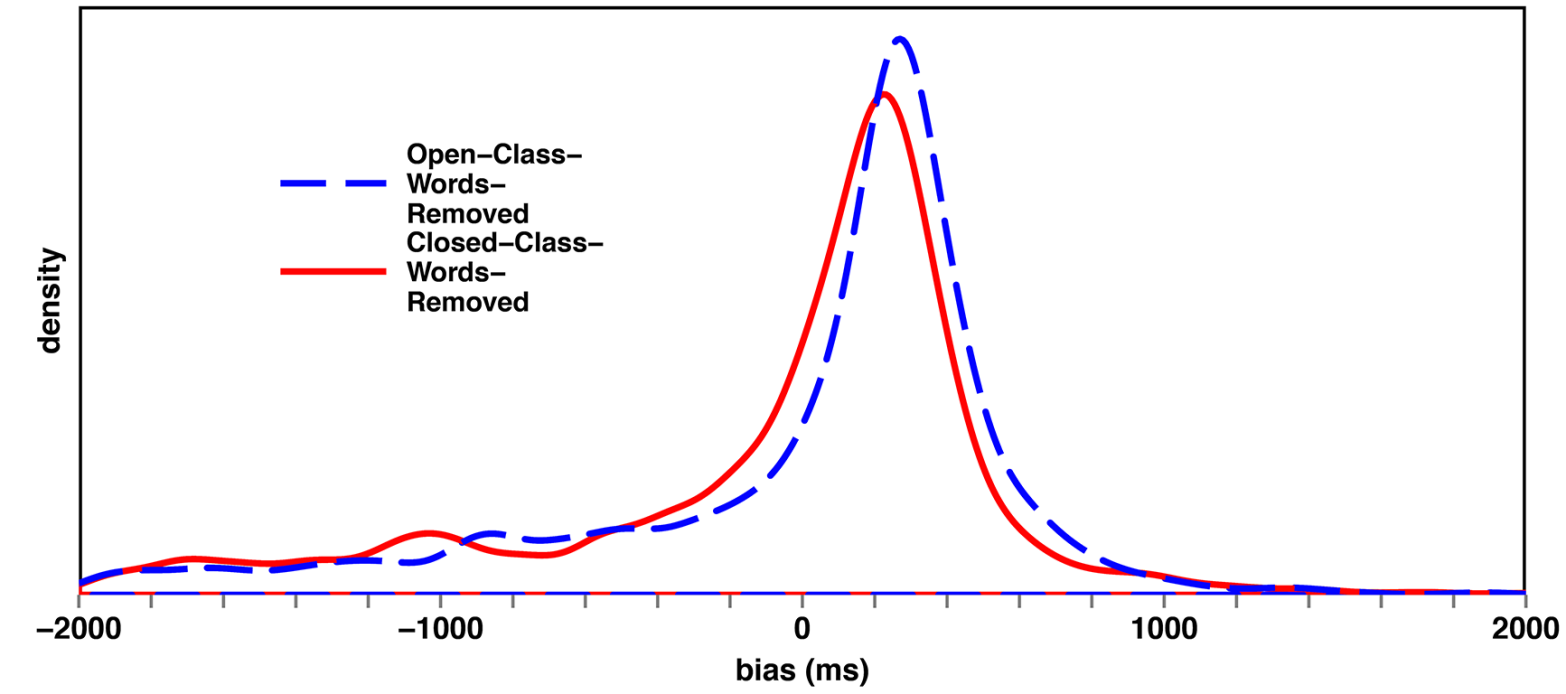
**Density plot of the BIAS distribution (in ms) of the Open-Class-Words-Removed and the Closed-Class-Words-Removed conditions.** A paired *t*-test revealed that the difference of the means of the two conditions was significant [*t*(99) = -3.23, *p* < 0.01].

The BIAS distributions of the critical conditions Closed-Class-Words-Removed and Open-Class-Words-Removed, shown in **Figure 9**, supported the RAP analysis.

The results showed that when closed class words are removed participants are still able to anticipate the turn ending, although compared to the Natural turn condition the anticipation performance deteriorated. But when the participants could only identify closed class words (and not open class words) they reacted significantly more frequently to the turn end than when only open class words were identifiably.

The results suggest that semantic information is more important than syntactic information for end-of-turn anticipation. If no semantic information is available, it is less likely for the listeners to anticipate the turn ending. This also means that only knowing the syntactic frame and number of slots is not sufficient to estimate the timing of the turn. Nevertheless, the anticipation performance increased significantly when both open class words as well as closed class words were available. This could be explained by the fact that by removing closed class words the prediction of the content of the turn is also hampered. So for maximal anticipation performance listeners need semantic as well as syntactic information, probably because they need to be able to project the content of the turn. These results support the findings of Magyari and De Ruiter (2012) and Magyari et al. (2014) that listeners project the content of the turn to be able to estimate its duration.

Another interesting finding is that participants’ anticipation performance was significantly higher when they got only closed class words compared to when only intonational and rhythmical information was available. This indicates that on top of the prosodic properties the syntactic structure provides additional anticipation cues.

Taken together these results suggest that semantic information is the most essential cue for anticipation. But to be maximally capable to anticipate a turn ending listeners need both semantic and syntactic information, since only the combination of both information sources allows for a correct projection of the content of the turn.

## GENERAL DISCUSSION

In this study we addressed three different questions. First we investigated whether the observed accuracy in natural turn-taking was primarily due to anticipation or reaction to signals. Second, we wanted to quantitatively estimate the relative contribution of anticipation and reaction processes to the observed distribution of floor transfer timings. Finally, we studied the relative contribution of semantic and syntax in the timing of turn transitions.

In Experiment 1 we conducted a button press experiment in which we manipulated the information necessary for anticipation. The results showed that the listeners’ response accuracy and consistency are similar when they (a) heard the natural turn and (b) when they are maximally able to anticipate the turn end by having advance information about the turn content. We conclude that listeners are indeed able to anticipate a turn ending andthat they use this strategy consistently to tell when a turn is going to end. Thus, our findings support the functioning of the turn-taking mechanism proposed by Sacks et al. (1974). But it appears plausible that reaction to the turn ending could function as a “backup” mechanism in case of failures to anticipate turn-endings timely.

The data collected in Experiment 1 allowed us to estimate an empirical distribution for pure anticipation, sowe proceeded to assess the counterpart distribution for pure reaction in Experiment 2 by explicitly instructingparticipants to react to the end of noise signals. We combined the two distributions to estimate the RAP, whichrepresents the relative probability for a turn transition to have been guided by anticipation or reaction. By instructing the participants to react to the offset of a noise signal we estimated the ‘other extreme’ of anticipation, namely responding to the very end of a stimulus. We assume that a reaction to the offset of noise and a reaction to possible signals occurring at the very end of the turns (such as intonational patterns occurring immediately before the end of turns) are comparable from a reaction time point of view. It should bepointed out that it is also possible that conversationalists react to signals that occur before the very end ofthe turn. Because in our approach we assessed only the extreme opposites of pure (in the sense of ‘as pure as practically achievable’) anticipation and reaction, our data do not allow for an estimation of the possible contribution of such responses to the relative proportion of anticipation and reaction.

In Experiment 3 we investigated the effect of the presence or absence of semantic and syntactic information on the anticipation and reaction probability using the RAP measure. The results showed that the participants werestill able to anticipate the end of the turn when they got access to semantic information. With only syntactic information available, the participants started to rely more on reaction. However, we found that to be maximallyable to anticipate, listeners need syntactic information as well as semantic information. The absence of syntactic information hampers the projection of the content of the turn. We concluded that for anticipation both semantic and syntactic information are needed. Nevertheless, it appears that semantic information is a more important cue than syntactic information.

The RAP measure introduced in Experiment 2 is not only an analysis tool for the characterization of turn transitions but implies an inherently *stochastic* view of the turn taking process. By empirically estimating, for the first time, separate probability distributions for anticipation and reaction processes in end-of-turn detection, we were able to estimate the relative probability for a turn transition to be caused by anticipation or reaction at a given FTO value. This differs from the approach by Heldner and Edlund (2010) who suggested that any FTO larger than 200 ms could plausibly be explained by reaction, while FTOs shorter than this threshold indicate anticipation. The latter approach does not allow for the realistic possibility that anticipation could have been late, or reaction could have been early. Our RAP measure provides this information and allows for a more realistic assessment of the individual role of anticipation and reaction in turn taking. In addition, our model makes it possible to address many open questions in turn taking research, especially regarding the mechanism itself and its robustness. Finally, a very exciting (though time-consuming) possibility is to derive RAP/FTO curves for different languages. The RAP could reflect differences in the timing of how different languages deliver discourse-relevant information. Here, morphosyntactic differences between languages, for instance languages with relatively free word order relying heavily on case marking versus languages like English with relatively fixed word order and a lean case marking system, may be reflected in different RAP/FTO curves. Alternatively, very similar RAP/FTO curves may suggest the presence of universals in the delivery of information in natural dialog.

Despite the mentioned advantages of the RAP measure over the strict threshold value suggested by Heldner and Edlund (2010), the RAP measure also does not incorporate the possibility of an intentionally delayed turn, for instance when that turn constitutes a ‘dispreferred response.’ Although this can be shown to happen in natural conversations, it is a situation that is difficult to recreate in a button press experiment; in our experimental setting we instructed the participants either to press the button when they thought the turn finished, or when the sound fragment was over. In this situation, we could not give the participants an interactional reason to delay their responses.

We showed in Experiment 3 that semantic information is a more important cue for anticipation than syntax. This finding contradicts former studies (Sacks et al., 1974; Selting, 1996; Caspers, 2003; De Ruiter et al., 2006) which assume that listeners rely primarily on syntactic information for anticipation. But how could semantic information serve to enable listeners to anticipate the turn ending? One possibility is that listeners use semantic information to predict the content of the speaker’s turn and thus are able to estimate which words will be produced to convey the content. This is in line with the findings of Magyari and De Ruiter (2012) and Magyari et al. (2014) that listeners are able to predict the upcoming words of a turn. Another possibility is that during their experience as conversationalists, listeners have over the years built up certain expectations about how much (new) semantic information, on average, a conversational turn tends to contain. If the amount of semantic information exceeds this expected amount, this could be exploited as a cue that the turn is about to end soon.

Another explanation for the importance of semantic information in turn-taking could be that in naturalistic contexts, the semantics may provide stricter constraints on the turn construction than syntax does. Syntax theoretically allows for an infinite extension of a turn by the addition of new constituents. Furthermore, non-sense sentences like the famous “Colorless beautiful green ideas sleep furiously” (Chomsky, 1957, p. 15) are syntactically correct but provide no reliable meaning to base anticipation on. In other words, the end of a “Jabberwocky” sentence is impossible to predict.

By presenting isolated turns from natural conversations and letting the participants respond to the end of the turn by a button press we could both keep the characteristics of natural speech and at the same time systematically manipulate the turn fragments in order to test our specific hypotheses. Nevertheless, by isolating the turns we are not able to consider the impact of dialog context on anticipation. The discourse context could add information about the speaker’s illocutionary intentions in the turn that is being produced, which in turn could help the listener anticipate its content. It is an interesting issue for future research whether, and if so, how, the discourse context can improve the anticipation of a turn ending.

## AUTHOR CONTRIBUTIONS

The authors contributed to the following activities. Carina Riest and Jan P. de Ruiter designed the studies. Carina Riest and Annett B. Jorschick collected the data. Carina Riest, Annett B. Jorschick, and Jan P. de Ruiter analyzed the data. The paper was drafted by Carina Riest and revised by Annett B. Jorschick and Jan P. de Ruiter. All authors approved the final version of the manuscript and agreed to be accountable for all aspects of the work.

## Conflict of Interest Statement

The authors declare that the research was conducted in the absence of any commercial or financial relationships that could be construed as a potential conflict of interest.

## Footnotes

1 In order to be able to meaningfully compare the higher moments of the two distributions, three outliers containing unrealistically large positive FTO values were removed from the data from the experiment by De Ruiter et al. (2006) for this table.

2 We are aware that open class words can contain syntactic cues, such as inflections and agreement on verbs and case marking on nouns, and closed class words can also contain referential information. Particularly in the Closed-Class-Words condition information as inflections could have helped to better anticipate the end of the turn.
